# Effect of Maternal *Schistosoma mansoni* Infection and Praziquantel Treatment During Pregnancy on *Schistosoma mansoni* Infection and Immune Responsiveness among Offspring at Age Five Years

**DOI:** 10.1371/journal.pntd.0002501

**Published:** 2013-10-17

**Authors:** Robert Tweyongyere, Peter Naniima, Patrice A. Mawa, Frances M. Jones, Emily L. Webb, Stephen Cose, David W. Dunne, Alison M. Elliott

**Affiliations:** 1 Department of Veterinary Pharmacy, Clinical and Comparative Medicine, Makerere University, Kampala, Uganda; 2 Uganda Virus Research Institute, Entebbe, Uganda; 3 Department of Pathology, Cambridge University, Cambridge, United Kingdom; 4 London School of Hygiene and Tropical Medicine, London, United Kingdom; Liverpool School of Tropical Medicine, United Kingdom

## Abstract

**Introduction:**

Offspring of *Schistosoma mansoni*-infected women in schistosomiasis-endemic areas may be sensitised *in-utero*. This may influence their immune responsiveness to schistosome infection and schistosomiasis-associated morbidity. Effects of praziquantel treatment of *S. mansoni* during pregnancy on risk of *S. mansoni* infection among offspring, and on their immune responsiveness when they become exposed to *S. mansoni*, are unknown. Here we examined effects of praziquantel treatment of *S. mansoni* during pregnancy on prevalence of *S. mansoni* and immune responsiveness among offspring at age five years.

**Methods:**

In a trial in Uganda (ISRCTN32849447, http://www.controlled-trials.com/ISRCTN32849447/elliott), offspring of women treated with praziquantel or placebo during pregnancy were examined for *S. mansoni* infection and for cytokine and antibody responses to SWA and SEA, as well as for T cell expression of FoxP3, at age five years.

**Results:**

Of the 1343 children examined, 32 (2.4%) had *S. mansoni* infection at age five years based on a single stool sample. Infection prevalence did not differ between children of treated or untreated mothers. Cytokine (IFNγ, IL-5, IL-10 and IL-13) and antibody (IgG1, Ig4 and IgE) responses to SWA and SEA, and FoxP3 expression, were higher among infected than uninfected children. Praziquantel treatment of *S. mansoni* during pregnancy had no effect on immune responses, with the exception of IL-10 responses to SWA, which was higher in offspring of women that received praziquantel during pregnancy than those who did not.

**Conclusion:**

We found no evidence that maternal *S. mansoni* infection and its treatment during pregnancy influence prevalence and intensity of *S. mansoni* infection or effector immune response to *S. mansoni* infection among offspring at age five years, but the observed effects on IL-10 responses to SWA suggest that maternal *S. mansoni* and its treatment during pregnancy may affect immunoregulatory responsiveness in childhood schistosomiasis. This might have implications for pathogenesis of the disease.

## Author Summary

Infections with the blood fluke *Schistosoma mansoni* that cause schistosomiasis (also called Bilharzia) were not usually treated during pregnancy until 2002, but in 2002 a World Health Organization (WHO) team of experts recommended that praziquantel treatment of *S. mansoni* during pregnancy should be done. However, there was limited information on the effects of maternal *S. mansoni* infection and treatment during pregnancy on the outcomes in the offspring. We conducted a study in the Entebbe peninsula within Lake Victoria in Uganda to examine whether maternal *S. mansoni* infection or its treatment during pregnancy may have effects on the children's susceptibility to the infection. The children were examined at age five years old for the level of *S. mansoni* infection and for immune responses to schistosomes. At five years old few of the children in our study cohort were infected with *S. mansoni*. Our findings suggest that maternal infection with, or praziquantel treatment of S. mansoni during pregnancy did not influence the level of *S. mansoni* infection among the offspring. However our findings suggest an influence on regulation of the body's immune responses to schistosomes, which may have some effect on the progress of disease manifestations. This is an issue that needs further investigation.

## Introduction

*In-utero* sensitisation to schistosome antigens occurs in up to 50% of offspring born to women with schistosomiasis during pregnancy [1], [2]. Those who do not show an immune response may not have been exposed to relevant antigen (due to the placental barrier) or may have been exposed and developed tolerance. For those who are tolerant, this may be due to induction of regulatory mechanisms, or to deletion of schistosome antigen specific cells. When the baby is sensitised *in-utero*, profiles of infant responses resemble the mothers' responses: mixed type one, type two cytokine responses and a prominent IL-10 response, as shown by our recent study on cord blood responses to schistosome worm (SWA) and egg (SEA) [3] and one earlier study [2]. Our study suggested a direct correlation between maternal *S. mansoni* infection intensity and cord blood T cell sensitisation of the offspring [3]. Thus immune responses to schistosome antigens typical of those observed in adults can be established *in-utero* in offspring born to schistosome-infected women.

In communities where schistosomiasis is endemic, an age-dependent build up of immune response and resistance to *S. mansoni* infection has been described [4], [5]. There is a possibility that, in addition to this, immune responses established *in-utero* might influence early childhood responses and resistance to infection. On the other hand, children who are exposed *in-utero*, but do not become sensitised, may have acquired tolerance to schistosome antigens, which might render them more susceptible to infection, as has been observed among offspring of women with filariasis [6]. *In-utero* sensitisation to schistosome antigens might also influence schistosomiasis morbidity. For example, the low incidence of the acute schistosomiasis syndrome, Katayama fever [7], in endemic populations is attributed to early or *in-utero* sensitisation such that they are able to modulate the immune response and subsequent pathology associated with *S. mansoni* infection.

Balanced type one/type two immune responses are needed to limit morbidity [8], [9], [10], [11], [12], and responses established *in-utero* could influence this balance. Studies in mice support the hypothesis that *in-utero* anti-idiotypic exposure induces B and T cell responsiveness to schistosome antigens recognised by the idiotype [13], and that this exposure induces immmunoregulatory effects in subsequent schistosomiasis infection [14]. Recent immunoepidemiological studies in Kenya involving school age children (4–17 years) in *S. mansoni* endemic areas showed that regulatory cytokines are important in schistosomiasis morbidity [15], and that dysregulation of pro-inflammatory cytokines could be a mechanism involved in childhood hepatosplenomegaly observed in schistosomiasis.

It is estimated that in Africa up to 10 million women per year have schistosomiasis during pregnancy [16], and 40 million women of child-bearing age have schistosomiasis [17]. Here we report results on follow-up of offspring at five years old, exploring the hypothesis that schistosomiasis and its treatment during pregnancy could influence the immune responses of the children when they themselves are exposed to *S. mansoni* infection. Based on the immunoepidemiological studies that explored association of the various cytokines with *S mansoni* morbidity [15], [18], [19], we measured IFN, IL-5, IL-13 and IL-10 responses to schistosome worm (SWA) and egg (SEA) antigens and regulatory T-cells.

## Methods

### Ethics statement

Written informed consent was obtained from each participant [20]. For all the children's participation, written informed consent was obtained from their parent or guardian. Ethical approval was obtained from the Science and Ethics Committees of the Uganda Virus Research Institute – Ministry of Health, the Uganda National Council for Science and Technology and the London School of Hygiene and Tropical Medicine.

### Study setting, subjects and sample collection

This study was a follow-up of children within the ‘Entebbe Mother and Baby Study’ (EMaBS; ISRCTN 32849447) [20]. EMaBS was a randomised, double-blind placebo-controlled trial of praziquantel versus matching placebo and albendazole versus matching placebo during pregnancy using a 2×2 factorial design. It was conducted within Entebbe municipality and the adjacent Katabi sub-county in Uganda. Previous policy had excluded pregnant and lactating women from the control of schistosomiasis using praziquantel treatment [21], and although this policy was rescinded [22] there had been limited information on the effects of praziquantel treatment of schistosomiasis during pregnancy regarding anticipated benefits (on outcomes such as anaemia and birth weight) or possible adverse effects on the developing fetus. Hence there was considered to be the required equipoise to justify placebo-controlled trials [23]. Pregnant women were recruited between April 2003 and November 2005. At recruitment, the women provided stool samples that were examined for intestinal helminths including *S. mansoni* infection using the Kato Katz method [24]. Children were followed up annually and stool and blood samples were collected at each visit. For the current study, we focussed on offspring of the study women at age five years.

The stool samples were examined for helminth infections including *S. mansoni* by the Kato Katz method for all children. Blood samples (approximately 6 mL) were processed for immune responses, plasma was separated and stored at −80°C, and peripheral blood mononuclear cells (PBMCs) were isolated and stored in liquid nitrogen. For schistosome immunology studies, whole blood culture for responses to schistosome antigens, and anti-schistosome antibody assays, were performed in a subgroup of 436 children, comprising 190 whose mothers had *S. mansoni* infection during pregnancy and, for comparison, a consecutive series of 246 children whose mothers did not.

### Whole blood culture and cytokine assay

Whole blood stimulation for cytokine responses to SWA and SEA was performed as previously described [3], [25], [26], [27]. SWA and SEA antigens were supplied by Professor David Dunne (Cambridge University, UK). The levels of endotoxin in the antigen preparations were 0.086 EU/mg for SWA and 0.175 EU/mg for SEA. On dilution of the antigen to working concentration of 10 µg/ml, the endotoxin levels in culture were negligible (<0.1 ng/ml) and unlikely to influence cytokine responses in whole blood culture. Briefly, heparinised blood was diluted 1 in 4 with serum-free medium (RPMI supplemented with glutamine, penicillin and streptomycin) and stimulated with SWA, SEA or phytohaemagglutinin (PHA- Sigma, UK) at final concentrations of 10 µg/ml in 96-well round-bottomed cell culture plates (TC Microwell, NUNC A/S, Roskelde, Denmark), or left unstimulated. Media (200 µl per well) was added to the plates and incubated at 37°C and 5% CO_2_. Supernatants were harvested after six days and left to stand at room temperature for one hour with viral inactivation buffer (0.03% tributyl phosphate and 1% Tween 80 (Sigma)) before storage at −80°C until needed for analysis.

The supernatants were assessed for interferon gamma (IFNγ), interleukin (IL)-5, IL-13 and IL-10 responses to SWA and SEA. IFNγ, IL-5 and IL-10 concentration in the supernatants was measured using OptEIA ELISA Kits (BD Pharmingen, USA) while IL-13 was measured using antibody pairs (BD Pharmingen, USA), with standards from the National Institute for Biological Standards and Controls (NIBSC, UK). The sensitivity of each assay, and cut-off for detectable responses, was the lowest concentration on the standard curve (9 pg/ml for IFNγ, 8 pg/ml for IL-5 and IL-10 and 16 pg/ml for IL-13). Cytokine levels in unstimulated cultures were generally low (Figure S1). To obtain antigen-specific responses, cytokine concentrations in unstimulated wells were subtracted from the measured concentrations in antigen-stimulated wells.

### Assay of antibodies to schistosome antigens

Plasma levels of IgG1, IgG4 and IgE to SWA and SEA were measured in duplicate by ELISA [24] with a high through-put semi-automated system. Briefly, flat bottomed 384-well microplates (Greiner Bio-One Ltd, Stonehouse, UK) were coated with SWA (8 µg/ml) or SEA (2.4 µg/ml) in 25 µl bicarbonate coating buffer and incubated overnight at 4°C. A standard positive pool and myeloma immunoglobulin isotype were added to allow a standard curve for quantification of antibodies to be generated. Samples were added at 25 µl per well at dilutions of 1/200 for IgG1 and IgG4 or 1/20 for IgE and incubated overnight at 4°C. Biotinylated mouse anti-human monoclonal antibodies (BD Pharmingen San Diego USA) were used for detection and Poly-HRP-streptavidin conjugate (Sanquin, Netherlands) was added at a 1/4000 dilution. Plates were developed with OPD substrate and stopped with 2 M sulphuric acid on observing the colour change. Optical densities (ODs) were recorded using Gen5 software. Concentrations were calculated from ODs by interpolation from standard curves. Normal European serum was included as a negative control to determine the cut-offs for positive levels. The cut-offs were set at three standard deviations above the mean of the normal European sera, which was 0.4 µg/ml for IgG1 to SWA, 0.1 µg/mL for IgG1 to SEA, 0.544 µg/ml for IgG4 to SWA, 0.042 µg/mL for IgG4 to SEA, 0.16 µg/mL for IgE to SWA and 0.04 µg/mL for IgE to SEA.

### Flow cytometry analysis of PBMCs for regulatory T Cells

PBMCs were stained for surface CD4, CD25, CD127 and intracellular FoxP3 to determine regulatory T cell (Treg) frequencies. Antibodies for surface marker staining were fluorescine isothiocyanate (FITC) labelled mouse anti-human CD4 (Invitogen-Caltag, Camarillo, USA) , phycoerythrin (PE) labelled mouse antihuman CD25 (Invitogen-Caltag, Camarillo, USA) and mouse anti-human CD127-Alexa647 (BD Pharmingen San Diego USA). Intracellular FoxP3 was detected using rat anti-human FoxP3-PE-cy5 (eBioscience, San Diego USA). PBMCs were thawed, washed and cell numbers determined. Between 1 and 2 million cells were stained in V-bottom 96-well plates. The cells were stained for 20 minutes in the dark at 4°C in 100 µl surface mix comprising surface antibodies CD4-FITC, CD25-PE and CD127-Alexa647 at dilutions 1∶40, 1∶20 and 1∶10 respectively. The cells were washed, permeabilized and stained for FoxP3 according to eBioscience's recommended protocol. The cells were acquired using FACS Diva software on a 17-colour flow cytometer (LSR II BD Biosciences) within 12 hours of staining. The acquired data was analysed using FlowJo software. Regulatory T cells (Tregs) were defined as CD4+ CD127low, CD25+ and FoxP3+. An example of the gating strategy applied can be viewed in Figure S2. The frequency of cells expressing FoxP3 was expressed as a percentage of CD4+ cells and the expression level of FoxP3 expressed in terms of geometric mean fluorescent intensity (GMFI).

### Statistical analysis

The analysis had four main objectives. (1) In the whole cohort, we examined whether *S. mansoni* infection during pregnancy was associated with childhood *S. mansoni* infection among the offspring at age five years. This was done using logistic regression to calculate odds ratios of infection among offspring at age five years, comparing prevalence among children of women who had *S. mansoni* infection during pregnancy with children of those who did not. For this observational analysis we also adjusted for factors that had a potential association with maternal *S. mansoni* infection in both mother and child, such as maternal residence, contact with lake water, maternal education, household socioeconomic status, maternal hookworm and *Mansonella* infections, using multivariable logistic regression. (2) In the whole cohort, we examined the effect of praziquantel treatment of *S. mansoni* infection during pregnancy on *S. mansoni* infection among the offspring at age five years. This was done by logistic regression comparing children of women that were treated, with children of women that were not treated. The possibility of a differential effect of maternal praziquantel treatment by maternal *S. mansoni* status was evaluated by conducting a subgroup analysis and calculating the p-value for the interaction. Since the prevalence of *S. mansoni* infection among children at age five years was very low, we further explored the consistency of the observed effect for objectives 1 and 2 using data collected at all annual visits, from age one year to age eight years, using repeated measures analysis combining available infection data from all time points. (3) In the subgroup selected for schistosome immunology studies, we examined associations between the schistosome-specific immune responses of the offspring at age five years and maternal *S. mansoni* infection during pregnancy, and associations between schistosome-specific immune responses in five year olds and praziquantel treatment during pregnancy. This was done by comparing cytokine and antibody responses to SWA and SEA between offspring of women who had *S. mansoni* infection during pregnancy and those that did not (restricting to the placebo group), and between children of women of the praziquantel and placebo groups (restricting to infected women). Cytokine and antibody responses had skewed distributions. The data were therefore log_10_ transformed and analysed by linear regression with bootstrapping to estimate bias-corrected accelerated regression coefficients and 95% confidence intervals that were back-transformed to give geometric mean ratios. Levels were also compared between infected and uninfected children using Wilcoxon rank sum test. (4) We examined the effect of maternal schistosomiasis and treatment during pregnancy on regulatory T cells in the offspring. This was done by comparing the proportion of CD4+ cells that expressed FoxP3, and the levels of FoxP3 expression, between children of women with schistosomiasis and those without, and also between children of infected women that were treated or not. Similarly, these comparisons were done by linear regression with bootstrapping to estimate bias-corrected accelerated regression coefficients and 95% confidence intervals that were transformed to give geometric mean ratios.

## Results

### Baseline characteristics of the study cohort

Of the 2507 women enrolled in the main study, 2345 live births were recorded and 1343 of these children were examined for *S. mansoni* at age five years, 703 in the placebo group and 640 in the praziquantel group. The baseline characteristics of the mothers as recorded at enrolment into the study during pregnancy did not significantly differ between the praziquantel and placebo arms (Table 1). Among the placebo group (n = 703), 571 (81.2%) were uninfected, 85 (12.1%) had light, 24 (3.4%) moderate and 23 (3.3%) heavy infection. Among the praziquantel group (n = 640), 519 (81.1%) were uninfected, 80 (12.5%) had light, 23 (3.6%) moderate and 18 (2.8%) heavy infection. Thus of the 1343 children, 253 (132 placebo, 121 praziquantel) were of women that had *S. mansoni* during pregnancy and 1090 (571 placebo, 519 praziquantel) were of uninfected women. Women that had *S. mansoni* at enrolment were less likely to live in Entebbe town and more likely to have regular contact with lake water (Table 1).

**Table 1:** Baseline characteristics at enrolment (during pregnancy) for the mothers of the children examined at age 5 years.

	Placebo (n = 703)		Praziquantel (n = 640)	
	Mother had S. mansoni	Mother uninfected	Mother had S. mansoni	Mother uninfected
	(n = 132 (18.8%))	(n = 571)	(n = 121 (18.9%))	(n = 519)
Maternal helminth infections				
Hookworm	52 (39.4%)	230 (40.3%)	48 (39.7%)	231 (44.5%)
Mansonella perstans	20 (15.2%)	114 (20.0%)	12 (10.0%)	125 (24.1%)
Trichuris	21 (15.9%)	40 (7.0%)	15 (12.4%)	42 (8.1%)
Ascaris	2 (1.5%)	14 (1.5%)	8 (6.6%)	8 (1.5%)
Any worm	132 (100%)	314 (55.0%)	121 (100%)	308 (59.0%)
Mother education				
None	8 (6.1%)	13 (2.3%)	2 (1.7%)	16 (3.1%)
Primary	66 (50.0%)	281 (49.3%)	67 (55.4%)	255 (49.2%)
Secondary	50 (37.9%)	226 (39.7%)	46(38.0%)	192 (37.1%)
Tertiary	8 (6.1%)	50 (8.8%)	6(5.0%)	55 (10.6%)
House hold socioeconomic status				
1(low)	5 (3.8%)	35 (6.1%)	7 (5.8%)	32 (6.2%)
2	11 (8.3%)	41 (7.2%)	10 (8.3%)	36 (6.9)
3	42 (31.8%)	153 (26.8%)	30 (24.8%)	160 (30.8%)
4	45 (34.1%)	167 (29.3%)	43 (35.5%)	143 (27.6%)
5	21 (15.9%)	129 (22.6%)	21 (17.4%)	104 (20.0%)
6(high)	5 (3.8%)	36 (6.3%)	7 (5.8%)	36 (6.9%)
Missing the information	3 (2.3%)	10 (1.8%)	3 (2.5%)	8 (1.5%)
Location				
Entebbe town	38 (28.8%)	237 (41.5%)	34 (28.1%)	194 (37.4%)
Kigungu	22 (16.7%)	65 (11.4%)	21 (17.4%)	66 (12.7%)
Manyago	46(34.9%)	148 (25.9%)	42 (34.7%)	145 (27.9%)
Katabi road side	9 (6.8%)	71 (12.4%)	6 (5.0%)	70 (13.5%)
Katabi off road	16 (12.1%)	44 (7.7%)	17 (14.1%)	40 (7.7%)
Other	1 (0.8%%)	6 (1.1%)	1 (0.8%)	4 (0.8%)
Mother Lake water contact				
Never	36 (27.3%)	211 (37.0%)	24 (19.8%)	199 (38.3%)
Rarely	38 (28.9%)	150 (26.3%)	39 (32.2%)	112 (21.6)
Weekly	9 (6.8%)	29 (5.1%)	13 (10.7%)	22 (4.2)
Daily	11 (8.3%)	33 (5.8%)	13 (10.7%)	31 (6.0%)
Missing the information	38(28.8%)	148 (25.9%)	32 (26.5%)	155 (29.9%)

### *S. mansoni* infection among children of the cohort at 5 years old

Overall, 32 of the 1343 children (2.4%) had *S. mansoni* infection at age five years based on a single stool sample. Of these 11 children (3 placebo group and 8 praziquantel group) were of women that had *S mansoni* infection during pregnancy while 21 (10 placebo group, 11 praziquantel group) were of uninfected women. Despite the few children infected, some had a high infection intensity with a geometric mean infection intensity of 106 eggs per gram (epg) of stool (minimum 12 epg, maximum 3384 epg). Fifteen (45.9%) had light (<100 epg), twelve (37.5%) had moderate (100–399 epg) and five (15.6%) had heavy (>400 epg) infections [21]. Among the children with *S mansoni* in the placebo group (n = 13), 5 had light, 6 moderate and 2 heavy infection while among those in the praziquantel group (n = 19), 10 had light, 6 moderate and 3 heavy *S. mansoni* infection.

Soil transmitted helminth seen among the children at age 5 years were hookworm 6 (0.4%), *Ascaris* 13 (1.0%), and *Trichuris* 73 (5.5%) and were mainly among the children with no *S. mansoni* infection. No children with *S mansoni* had hookworm or *Ascaris* and four children had co-infection of *S mansoni* and *Trichuris*.

We explored the association of factors including maternal helminth infections and treatment, location of residence, contact with lake water, maternal education, and household socioeconomic status (a score based on the house building materials, number of rooms and items owned), with the presence of *S. mansoni* infection in the children. As expected, contact with lake water and location of residence were associated with the children having *S. mansoni* infection (Table 2). Children whose mothers who resided in Kigungu or Katabi village (lakeside locations) were more likely to have *S. mansoni* infection than those from Entebbe town, which is not directly on the lakeshore. It was also noted that the proportion of children that reported having been ‘in contact with lake water frequently’ was greater for Kigungu (38.5%) and Katabi village (24.8%) than Entebbe town (14.5%), Katabi town (6.9%) or Manyago (11.5%) (P<0.001). Although crude analysis suggested that maternal *S. mansoni* infection was associated with *S. mansoni* infection among the children at five years old, on adjusting for the other factors (location of residence, contact with lake water, maternal education, and household socioeconomic status) this association was lost. Trial analysis showed that praziquantel treatment of the women during pregnancy had a tendency to increase the chances of their offspring being infected (Table 3), although this was not statistically significant. The effect of praziquantel treatment appeared somewhat stronger among children of mothers with *S. mansoni*, although again it was not significantly different to the effect of treatment among children of uninfected mothers (interaction p = 0.249). Because the prevalence of *S. mansoni* infection was low at five years, we also explored for effects of maternal praziquantel treatment during pregnancy on prevalence of *S. mansoni* infection among the study cohort children using a repeated measures analysis combining all data from annual time points to date (comprising age one to six for the youngest, and one to eight years for the oldest children, depending on the child's age at the time of the analysis). Again, no association was observed OR 0.80 (95% CI 0.57, 1.13 p = 0.21).

**Table 2:** Factors associated with *S. mansoni* infection among children at age five years old.

Variable	*S. mansoni* prevalence among the children at age five years	Crude OR (95%CI)	Multivariable adjusted OR* (95% CI)	Likelihood ratio p-value
Maternal *S. mansoni* infection				
Un infected (n = 1090)	1.90%	1	1	
Infected (n = 253)	4.40%	2.3 (1.1, 4.9)	1.1 (0.4, 3.3)	0.796
Location				
Entebbe town (n = 503)	0.80%	1	1	
Kigungu (n = 174)	6.30%	8.4(2.6, 26.8)	4.5 (0.8, 25.0)	
Manyago (n = 381)	1.30%	1.7 (0.4, 6.2)	3.3 (0.5, 22.3)	
Katabi town (n = 156)	0%	-	-	
Katabi village (n = 117)	10.30%	14.3 (4.5, 45.1)	18.6 (3.3, 104.2)	0.002
Maternal contact with lake water				
Never (n = 470)	0.60%	1	1	
Rarely (n = 339)	2.10%	3.3 0.8, 12.8)	1.0 (0.2, 6.8)	
Weekly (n = 73)	5.50%	9.0 (2.0, 41.2)	1.8 (0.2, 15.5)	
Daily (n = 88)	13.60%	24.6 (6.8, 89.1)	3.7 (0.5, 27.1)	0.282
Maternal Socioeconomic status				
V. Poor (n = 177)	4.50%	1	1	
Poor (n = 385)	2.60%	0.6 (0.2, 1.5)	0.9 (0.2, 3.3)	
Fair (n = 398)	2.00%	0.4 (0.2, 1.2)	0.8 (0.2, 2.9)	
Better (n = 359)	1.40%	0.3 (0.1, 0.9)	0.3 (0.04, 1.9)	0.533
Maternal Education				
None (n = 39)	2.60%	1	1	
Primary (n = 669)	3.00%	1.2 (0.2, 9.0)	0.5 (0.1, 5.5)	
Secondary (514)	1.80%	0.7 (0.1, 5.5)	0.2 (0.02, 2.8)	
Tertiary (119)	1.70%	0.6 (0.1, 7.4)	0.3 (0.01, 12.4)	0.474
Child contact with lake water				
Never (n = 486)	0.40%	1	1	
Rare (n = 195)	4.60%	11.7 (2.5, 54.7)	7.1 (1.3, 40.7)	
Weekly (n = 41)	9.80%	26.2 (4.6, 147.6)	7.9 (1.0, 63.5)	
Daily (n = 46)	19.60%	58.9 (12.3, 282.4)	21.4 (3.0, 151.0)	0.006

* Multivariable analysis odds ratios adjusted for variables presented in the table as well as maternal hookworm and microfilaria infection.

**Table 3:** Effect of praziquantel treatment during pregnancy on *S. mansoni* infection status in children at age five years.

Treatment during pregnancy	Children with *S. mansoni* (%)	OR (95% CI) p value
Overall*		
Placebo (n = 703)	13 (1.9%)	1
Praziquantel (n = 640)	19 (3.0%)	1.6 (0.80, 3.32) p = 0.183
**Mother had ** ***S mansoni***		
Placebo (n = 132)	3 (2.3%)	1
Praziquantel (n = 121)	8 (6.6%)	3.0 (0.79, 11.75) p = 0.106
**Mother uninfected**		
Placebo (n = 561)	10 (1.8%)	1
Praziquantel (n = 519)	11 (2.1%)	1.2 (0.51, 2.88) p = 0.659

### Cytokine responses to SWA and SEA among the children at age five years

Four hundred and thirty six children were examined for IFNγ, IL-5, IL-10 and IL-13 responses to SWA and SEA. Of these, 207 were the offspring of women who received praziquantel treatment during pregnancy (praziquantel group) and 229 were of women who did not receive the treatment (placebo group). Restricting our initial analysis to the placebo group, we examined whether cytokine responses to SWA or SEA among the children differed between children of *S. mansoni* infected women and those of uninfected women (Table S1 in Text S1). Maternal *S. mansoni* infection did not show any significant associations with cytokine responses among the children, with the exception of IL-10 responses to SWA; children of women that had *S. mansoni* during pregnancy had lower IL-10 responses to SWA (geometric mean (cytokine concentration +1) = 6.1 pg/mL) than children of uninfected women (geometric mean (cytokine concentration+1) = 3.5 pg/mL, geometric mean ratio 0.58, 95%CI 0.39, 0.90)). We examined for associations between cytokine responses and maternal infection intensity during pregnancy and found no significant correlation (data not shown).

**Figure 1:**
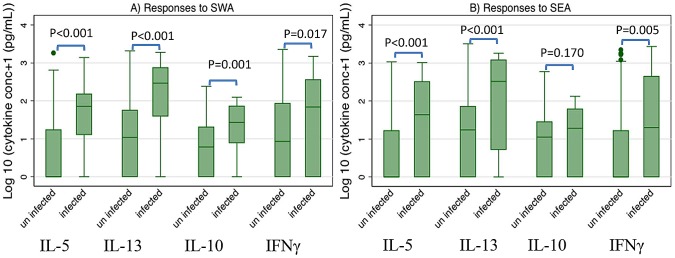
Cytokine responses to SWA (A) or SEA (B) among children at age five years. Comparisons are shown between uninfected children (n = 390) and infected children (n = 20). Presented are Wilcoxon rank-sum test p-values.

**Figure 2:**
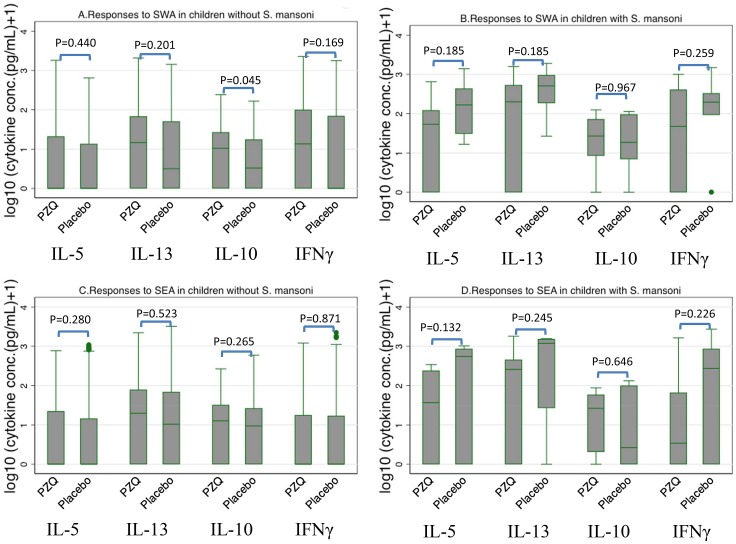
Effect of praziquantel treatment during pregnancy on cytokine responses to SWA or SEA among the children infected or not infected with *S mansoni* at age 5 years. Infected children, praziquantel group (PZQ) n = 14, placebo n = 6; uninfected children, PZQ n = 178, placebo n = 206. Shown are levels of cytokine production in response to SWA (A & B) or SEA (C &D).

**Figure 3:**
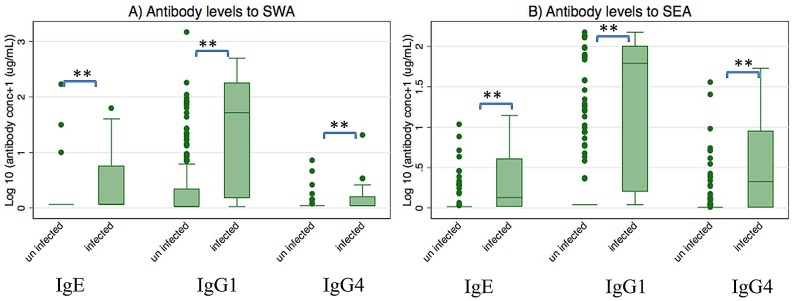
Antibody levels to SWA or SEA among children at age five years. Shown are plasma levels of antibodies to SWA (A) or SEA (B) among uninfected children (n = 390) and infected children (n = 20) at age five years. ** Wilcoxon rank-sum test p<0.001.

When we examined for effects of praziquantel treatment of *S. mansoni* during pregnancy on cytokine responses among the children, significant effects were only seen for IL-10 responses to SWA where the responses were higher among children of women who received praziquantel treatment during pregnancy (geometric mean (cytokine concentration +1) = 3.5 pg/mL) than those of women who were not treated (geometric mean (cytokine concentration +1) = 7.0 pg/mL, (geometric mean ratio 1.97, 95%CI 1.25, 3.23)). More information can be viewed in Table S2 in Text S1. Of the children examined for cytokine responses, 20 children had *S. mansoni* infection at age five years. As expected, cytokine responses were higher in children with *S. mansoni* than those without (Figure 1) and the cytokine profile among the children was a mixed type 1, type 2, and regulatory response. Among the uninfected children, praziquantel treatment during pregnancy did not influence cytokine responses to SWA or SEA except for IL-10, for which the responses to SWA were still higher among children of women who received praziquantel treatment compared with those of untreated women (geometric means 6.1 and 4.4 pg/mL, respectively geometric mean ratio 1.38 (95% CI 1.01, 1.90); Figure 2). The low number of infected children limited similar subgroup analysis among the infected children. Nevertheless, the responses among the few (4 placebo group, 16 praziquantel group) infected children at age five years presented a different picture from the responses among the uninfected children (Figure 3; compare A with B for SWA or C with D for SEA). Praziquantel treatment of the women during pregnancy showed a tendency to reduce IFNγ, IL-5, and IL-13 responses to SWA and SEA, but not IL-10 in infected children.

### Levels of antibodies to SWA and SEA among children at age five years

**Figure 4:**
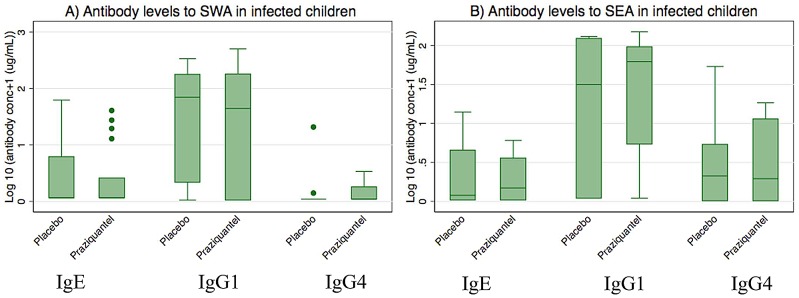
Effect of praziquantel treatment of women during pregnancy on levels of antibodies to SWA or SEA among their offspring having *S. mansoni* at age 5 years. Shown are plasma levels of antibodies to SWA (A) or SEA (B) among children that had *S mansoni* infection at age five years.

Levels of IgG1, IgG4 and IgE to SWA and SEA were measured in 226 children at age five years. Of these, 142 (63 placebo and 79 praziquantel group) were the off-spring of women infected with *S. mansoni* during pregnancy and 84 (54 placebo and 30 praziquantel group) of uninfected women. Among the 226 children, SWA-specific IgG1 antibodies were detectable in 79 (35.0%) children, with SWA-specific IgG4 and IgE antibodies detected in 12 (5.3%) children. SEA-specific IgG1, IgG4 and IgE antibodies were detectable in 56 (24.8%), 52 (23.0%) and 33 (14.6%) children respectively. Of the children examined for antibody responses, 28 had *S. mansoni* at age five years. As expected, antibody levels showed associations with the children's infection status. Of the 28 children that had *S .mansoni* infection, IgG1, IgG4 and IgE to SWA were detectable in 21 (75%), 7 (25%) and 9 (32.1%) of the children respectively, and IgG1, IgG4 and IgE to SEA were detectable in 21 (75%), 19 (67.9%) and 15 (53.6%) children, respectively. Levels of IgG1, IgG4 and IgE to SWA or SEA were minimal among uninfected children and were significantly lower than the levels among the *S. mansoni* infected children (Figure 3, p<0.001 for the respective comparisons). Maternal infection intensity was not associated with antibody levels (data not shown) and there were no significant effects of praziquantel treatment during pregnancy on antibody levels among the offspring (Figure 4).

### Expression of regulatory T cell (Treg) phenotype among the children at age five years

Considering that *S. mansoni* infection has been associated with developing a strong immune regulatory response early in the initial encounter between the host and parasite [28], we explored whether maternal infection or treatment during pregnancy may influence long-term Treg expression in the offspring. Two hundred and nine children at age five years were examined for CD4+ T cell expression of FoxP3. Of these, 128 were the offspring of women infected with *S. mansoni* during pregnancy and 81 of uninfected women. Of the infected women, 68 received praziquantel treatment and 60 did not. We examined for effects of *S. mansoni* infection and praziquantel treatment during pregnancy on FoxP3 expression among offspring of women that were not treated during pregnancy. Maternal *S. mansoni* infection was not associated with FoxP3 expression in the children at age five years (data not shown) and praziquantel treatment during pregnancy did not influence expression of FoxP3 among the children (Table 4). Of the 209 children examined for FoxP3 expression, only 17 were infected with *S. mansoni* at age five years. FoxP3 expression was higher among the *S. mansoni* infected children than the uninfected children (Figure S3). We examined for associations between FoxP3 Treg expression and cytokine responses and found no significant associations (data not shown). This was done on pooled data as well as separately according to the children *S. mansoni* infection status.

**Table 4:** Effect of praziquantel treatment during pregnancy on regulatory T cells expression among offspring at age five years.

	%CD4 expressing FoxP3		Level of FoxP3 Expression (Geometric mean fluorescence intensity (GMFI))	
	Geometric Means	Geometric mean ratio (95% CI)	Geometric Mean	Geometric mean ratios (95%CI)
Overall				
Placebo (n = 112)	3.8		534.5	
Praziquantel (n = 97)	3.6	0.61 (0.21, 1.76)	630.6	1.18 (0.93, 1.47)
Mother had *S. mansoni*				
Placebo (n = 60)	3.7		591.4	
Praziquantel (n = 68)	3.2	0.33 (0.09, 1.38)	586.2	0.99 (0.73, 1.34)
Uninfected children				
Placebo (n = 103)	3.7		521.6	
Praziquantel (n = 85)	3.7	0.97 (0.32, 3.13)	586.7	1.12 (0.87, 1.45)
Children with *S. mansoni*				
Placebo (n = 7)	4.7		755.2	
Praziquantel (n = 10)	2.7	0.01 (0, 0.33)	897.5	1.19 (0.41, 2.85)

## Discussion

This study explored the effects of *S. mansoni* infection and of praziquantel treatment during pregnancy on *S. mansoni* infection and immune responsiveness among the offspring at the age of five years. This is an age by which the children are expected to have had their first exposure to infection with *S. mansoni* through playing in lake water. We found no conclusive evidence of an effect of maternal schistosomiasis, or of its treatment during pregnancy, on susceptibility to *S. mansoni* infection in the children, or on their anti-schistosome antibody levels. Similarly, no effect on cytokine responses to schistosome antigens was observed in the children apart from IL-10 responses to SWA, for which children of infected mothers tended to show lower responses than children of uninfected mothers, and children of praziquantel-treated mothers showed higher responses than children of placebo-treated mothers.

In this study the prevalence of *S. mansoni* among the five years old children was 2.4%. This was based on a single stool sample, implying that a number of infected children could have been misclassified due to the low sensitivity of a single stool sample Kato Katz test [29], and that these numbers may represent an under estimate of the actual prevalence [29], [30]. The observed prevalence was less than what has been reported from epidemiological surveys involving preschool children in other areas on the shores of Lake Victoria [31], [32], [33]. These studies were different from our study in that they applied consecutive multiple stool sample Kato Katz analysis. Further, the Entebbe area, where our study was carried out, is relatively urbanised and developing rapidly, and has wide availability of treated piped water. It is thus expected to have relatively low *S. mansoni* transmission. However, we noted that even under such low transmission conditions, infected young children might have quite high infection intensities. Children who were resident in the peri-urban areas of Kigungu and off road areas of Katabi were more likely to be infected with *S. mansoni* than children in the urban centres of Entebbe town and Katabi, and this is a direct connection to the frequency of contact with lake water. This underscores the concern that in situations where younger children are exposed to potential sources of infection, they will acquire the infection at an early age. This has implications on schistosomiasis control programs that have tended to neglect preschool children [34].

This study found no evidence that maternal *S. mansoni* infection *per se* or maternal praziquantel treatment may predispose children to infection with *S. mansoni*. Others have reported that children born to women with filariasis [35], and those affected by placental malaria [36], are likely to be infected earlier with the respective parasites, an effect that has been attributed to the induction of tolerance in the offspring [6]. Considering that our study included very few children showing infection at age five years, there is a further need to explore this effect in an area of high *S. mansoni* transmission. However, repeated measures analysis showed consistent observation among the cohort children at ages one to eight years gave further evidence that maternal praziquantel of S. mansoni during pregnancy may not influence prevalence of infection among offspring.

Cytokine responses among the children had a mixed type 1/type 2/regulatory profile. The same cytokine profile was found among mothers of the children in this study cohort [27], and immunoepidemiological studies have also shown a mixed responses with strong regulatory cytokine phenotype [37], [38]. It was also noted that expression of FoxP3 was prominent among the *S. mansoni* infected children. Our findings show that, in *S. mansoni* endemic areas, the profile of T-cell responses typical to those in *S. mansoni* chronically infected adults may be established in early childhood. For the observational analyses, where we compare infected and uninfected children, we observed quite marked differences in the immune responses in the children. Considering that we applied a single stool sample Kato Katz for diagnosis, misclassification of infected children as uninfected might have weakened the differences observed. Therefore these results are probably a conservative estimate of the effects of infection on immune responses in the children.

Praziquantel treatment during pregnancy did not influence most of the assayed cytokine responses to SWA or SEA. This finding is consistent with our earlier report in which we did not find significant effects of praziquantel treatment during pregnancy on cytokine responses to schistosome antigens among the offspring, in either cord blood or at age one year [3]. Of some interest, however, was the IL-10 response to SWA. This was an exception in that it was low in the offspring of mothers with *S. mansoni* infection and was increased by praziquantel treatment during pregnancy. IL-10 is a regulatory cytokine, capable of switching off both type 1 and type 2 immune responses, and could possibly explain why we saw consistently low IFNγ, IL-5, and IL-13 responses among infected offspring of women who received praziquantel treatment during pregnancy compared to offspring of those who did not (Figure 3 B&C). This could have implications on *S. mansoni* associated morbidity among the offspring. For example, low regulatory and type 2 responses to schistosome antigens have been associated with hepatosplenomegaly in childhood schistosomiasis [15]. In addition, low levels of regulatory cytokines and correspondingly higher levels of type 2 cytokines have been associated with a high risk of peri-portal fibrosis in chronic *S. mansoni* [18], [39] and *S. japonicum* [40]. In *S. japonicum* infection pro-inflammatory cytokines including IL-1, IL-6 and TNF have been associated with morbidity outcomes [41]. On the other hand, immunosuppressive responses are presumed to allow establishment and prolong survival of the worms in the host, preventing elimination of the parasite while at the same time minimizing tissue damage [42], [43], [44]. In our study the observations on IL-10, a regulatory cytokine, were not reflective of the regulatory T-cell data in terms of FoxP3 expression. However, IL-10 responses were measured after *in vitro* antigen stimulation of whole blood cultures, and it is likely that sources other than T cells also contributed to IL10 production. The observed effect on IL-10 response may have implications on schistosomiasis-associated morbidity, and this need further immunological and morbidity studies

In conclusion, and considering that the low *S. mansoni* prevalence in this study was a major limitation, our findings show no evidence that maternal *S. mansoni* infection or its treatment during pregnancy has an influence on the prevalence of *S. mansoni* infection among the offspring at age five years. However, our findings do suggest that maternal *S. mansoni* and its treatment during pregnancy may affect some cytokine responses among *S. mansoni* infected offspring at age five years. The magnitude of such an effect and possible implications in childhood schistosomiasis need to be elucidated further by conducting a larger study involving greater numbers of infected children.

## Supporting Information

Figure S1 Cytokine levels in supernatants of unstimulated cultures.
(PPTX)

Figure S2 Example of the gating strategy applied for regulatory T cell. The gate for A) Lymphocytes, B) selection to eliminate duplicates, C) CD4+ cells gate selection, D) CD4+CD25 high CD127 low selection and E) FoxP3 expression in the CD25hiCD127lo gated cell population.
(PPTX)

Figure S3 Expression of FoxP3 among children infected or uninfected with *S. mansoni* at age 5 years. On the Y-axis is the log10 (geometric mean fluorescent intensity (GMFI)+1).
(PPTX)

Text S1 Shown in **table S1** is the association of maternal *S. mansoni* infection during pregnancy with cytokine responses to SWA or SEA among children (n = 229) at age five years, and in **table S2 is the** effect of praziquantel treatment of *S mansoni* during pregnancy on cytokine responses to SWA/SEA in offspring at age five years.
(DOC)
