# Supplementary figures and images for: Effect of Maternal Schistosoma mansoni Infection and Praziquantel Treatment During Pregnancy on Schistosoma mansoni Infection and Immune Responsiveness among Offspring at Age Five Years
Source: PLoS Negl Trop Dis. 2013 Oct 17;7(10):e2501. doi: 10.1371/journal.pntd.0002501 (PMC3798616; doi:10.1371/journal.pntd.0002501)

## Slide 1
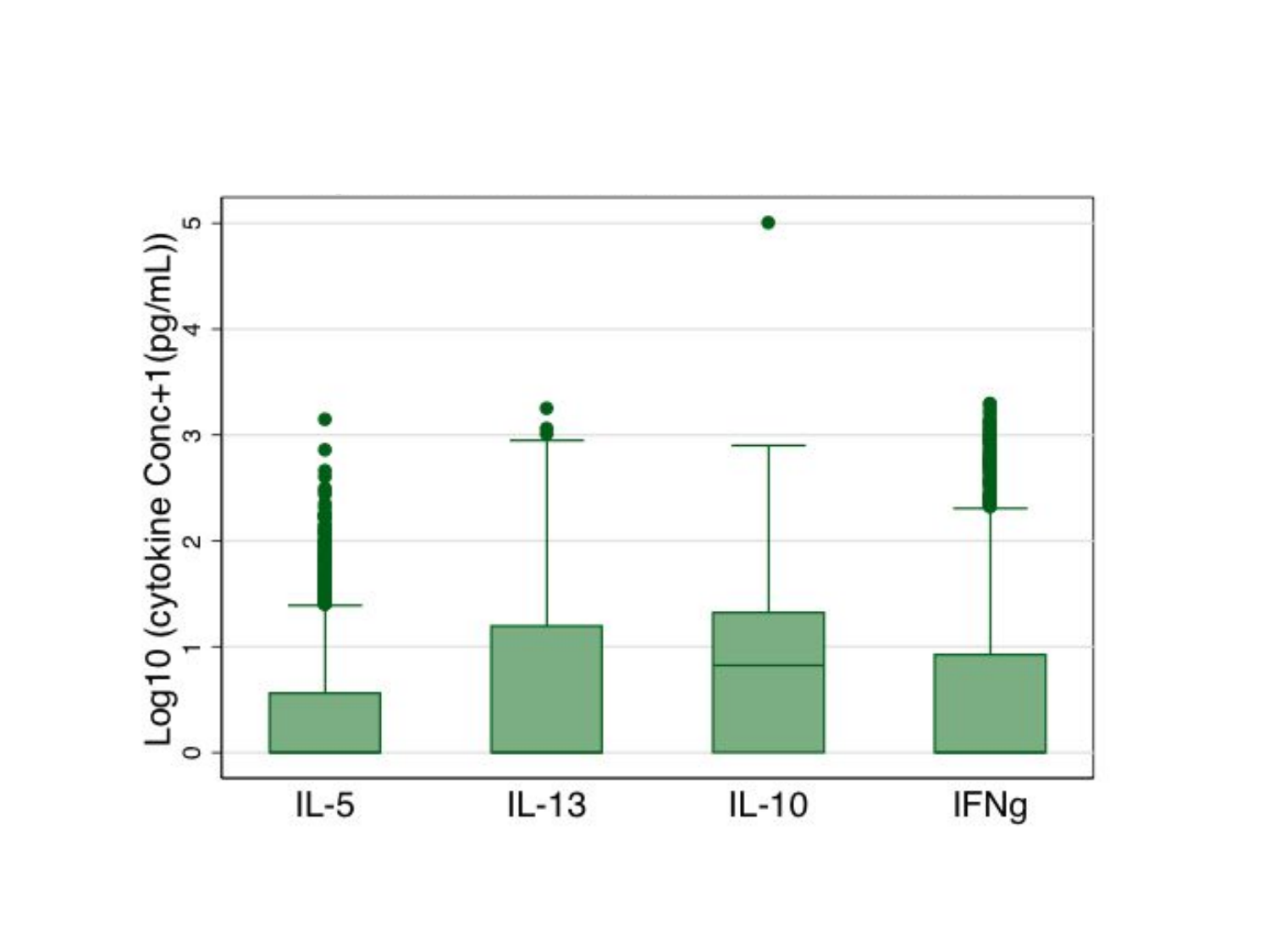

Supplement: Figure S1 — Cytokine levels in supernatants of unstimulated cultures. (PPTX) [file pntd.0002501.s001.pptx]

## Slide 1
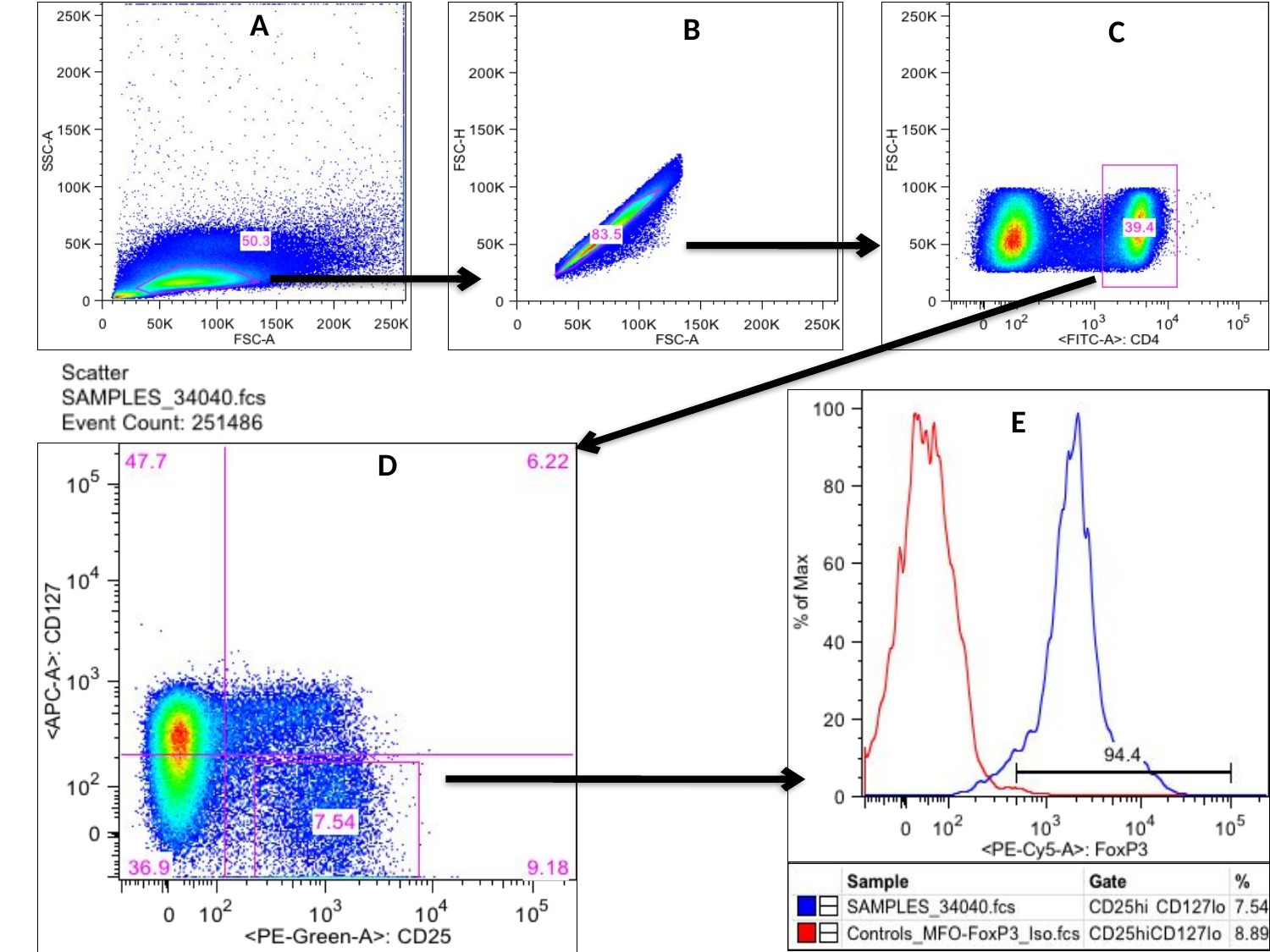

A
B
C
E
D

Supplement: Figure S2 — Example of the gating strategy applied for regulatory T cell. The gate for A) Lymphocytes, B) selection to eliminate duplicates, C) CD4+ cells gate selection, D) CD4+CD25 high CD127 low selection and E) FoxP3 expression in the CD25hiCD127lo gated cell population. (PPTX) [file pntd.0002501.s002.pptx]

## Slide 1
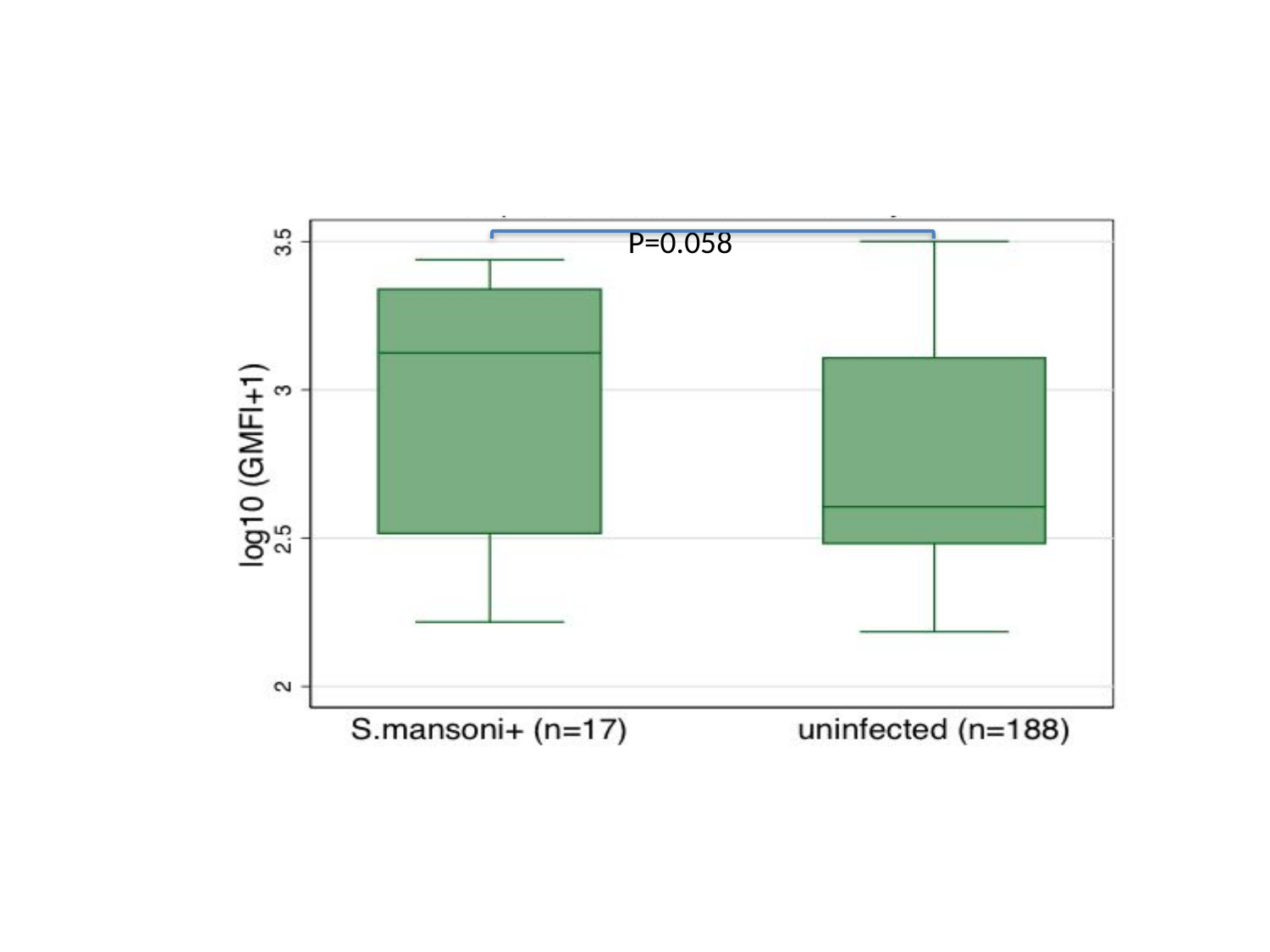

P=0.058

Supplement: Figure S3 — Expression of FoxP3 among children infected or uninfected with S. mansoni at age 5 years. On the Y-axis is the log10 (geometric mean fluorescent intensity (GMFI)+1). (PPTX) [file pntd.0002501.s003.pptx]
