# Supplementary material for: Effect of Maternal Schistosoma mansoni Infection and Praziquantel Treatment During Pregnancy on Schistosoma mansoni Infection and Immune Responsiveness among Offspring at Age Five Years
Source: PLoS Negl Trop Dis. 2013 Oct 17;7(10):e2501. doi: 10.1371/journal.pntd.0002501 (PMC3798616; doi:10.1371/journal.pntd.0002501)
Supplement: Text S1 — Shown in table S1 is the association of maternal S. mansoni infection during pregnancy with cytokine responses to SWA or SEA among children (n = 229) at age five years, and in table S2 is the effect of praziquantel treatment of S mansoni during pregnancy on cytokine responses to SWA/SEA in offspring at age five years. (DOC) [file pntd.0002501.s004.doc]

Table S1 Association of maternal *S. mansoni* infection during pregnancy with cytokine responses to SWA or SEA among children (n=229) at age five years

|  | Maternal *S. mansoni* status during pregnancy | Geometric mean of (cytokine concentration (pg/ML)+1) | Geometric mean ratio (95% CI) |
| --- | --- | --- | --- |
| Response to SWA |  |  |  |
| IFNγ | Uninfected (n=133) | 8.9 | 1 |
|  | Infected (n=94) | 10.2 | 1.15 (0.58, 2.16) |
| IL-5 | Uninfected (n=133) | 3.3 | 1 |
|  | Infected (n=94) | 4.5 | 1.39 (0.83, 2.35) |
| IL-13 | Uninfected (n=133) | 9.8 | 1 |
|  | Infected (n=94) | 8.1 | 0.82 (0.44, 1.52) |
| IL-10 | Uninfected (n=133) | **6.1** | **1** |
|  | Infected (n=94) | **3.5** | **0.58 (0.39, 0.90)** |
| Response to SEA |  |  |  |
| IFNγ | Uninfected (n=134) | 4.4 | 1 |
|  | Infected (n=95) | 4.4 | 1.00 (0.52, 1.81) |
| IL-5 | Uninfected (n=134) | 3.9 | 1 |
|  | Infected (n=95) | 3.8 | 0.98 (0.57, 1.81) |
| IL-13 | Uninfected (n=134) | 13.4 | 1 |
|  | Infected (n=95) | 8.7 | 0.66 (0.36, 1.33) |
| IL-10 | Uninfected (n=134) | 7.6 | 1 |
|  | Infected (n=95) | 5.6 | 0.75 (0.47, 0.82) |

Table S2. Effect of praziquantel treatment of *S mansoni* during pregnancy on cytokine responses to SWA /SEA in offspring at age five years

|  | Responses to SWA | | |  | Responses to SEA | | |
| --- | --- | --- | --- | --- | --- | --- | --- |
| Cytokine | Treatment of mother in pregnancy | Geometric mean of (cytokine conc.+1) (pg/mL) | Geometric mean ratio (95% CI) |  | Treatment of mother in pregnancy | Geometric mean of (cytokine conc. +1) (pg/mL) | Geometric mean ratio (95% CI) |
| IFNγ | Placebo (n=94) | 10.2 | 1 |  | Placebo (n=95) | 4.4 | 1 |
|  | Praziquantel (n=93) | 11.3 | 1.12 (0.53, 2.37) |  | Praziquantel (n=95) | 3.4 | 0.77 (0.42, 1.49) |
| IL-5 | Placebo (n=94) | 4.5 | 1 |  | Placebo (n=95) | 3.8 | 1 |
|  | Praziquantel (n=93) | 5.9 | 1.30 (0.73, 1.36) |  | Praziquantel (n=95) | 5.8 | 1.51 (0.79, 2.85) |
| IL-13 | Placebo (n=94) | 8.1 | 1 |  | Placebo (n=95) | 8.7 | 1 |
|  | Praziquantel (n=93) | 13.2 | 1.63 (0.80, 3,34) |  | Praziquantel (n=95) | 10.6 | 1.23 (0.56, 2.73) |
| IL-10 | Placebo (n=94) | 3.5 | 1 |  | Placebo (n=95) | 5.6 | 1 |
|  | Praziquantel (n=93) | 7 | **1.97 (1.25, 3.23)** |  | Praziquantel (n=95) | 8.1 | 1.44 (0.87, 2.28) |
